# Candidate variants in *TUB* are associated with familial tremor

**DOI:** 10.1371/journal.pgen.1009010

**Published:** 2020-09-21

**Authors:** M. Reza Sailani, Fereshteh Jahanbani, Charles W. Abbott, Hayan Lee, Amin Zia, Shannon Rego, Juliane Winkelmann, Franziska Hopfner, Tahir N. Khan, Nicholas Katsanis, Stefanie H. Müller, Daniela Berg, Katherine M. Lyman, Christian Mychajliw, Günther Deuschl, Jonathan A. Bernstein, Gregor Kuhlenbäumer, Michael P. Snyder

**Affiliations:** 1 Department of Genetics, Stanford University, Stanford, CA, United States of America; 2 Institute of Neurogenomics, Helmholtz Zentrum München, Munich, Germany; Institute of Human Genetics, Technical University, Munich, Germany; Munich Cluster for Systems Neurology (SyNergy), Munich, Germany; 3 Department of Neurology, Kiel University, Germany; 4 Department of Neurology, Hannover Medical School, Hannover, Germany; 5 Center for Human Disease Modeling, Duke University, United States of America; 6 Advanced Center for Translational and Genetic Medicine, Stanley Manne Children's Research Institute, Ann & Robert H. Lurie Children's Hospital, Chicago, IL, United States of America; 7 Department of Pediatrics, Feinberg School of Medicine, Northwestern University, Chicago, IL, United States of America; 8 Department of Neurology, Universitätsklinikum Tübingen, Germany; 9 University Hospital Tübingen, Department of Psychiatry and Psychotherapy, Tübingen, Germany; 10 Department of Pediatrics, Stanford University, Stanford, CA, United States of America; University of Pennsylvania Perelman School of Medicine, UNITED STATES

## Abstract

Essential tremor (ET) is the most common adult-onset movement disorder. In the present study, we performed whole exome sequencing of a large ET-affected family (10 affected and 6 un-affected family members) and identified a *TUB* p.V431I variant (rs75594955) segregating in a manner consistent with autosomal-dominant inheritance. Subsequent targeted re-sequencing of *TUB* in 820 unrelated individuals with sporadic ET and 630 controls revealed significant enrichment of rare nonsynonymous *TUB* variants (*e*.*g*. rs75594955: p.V431I, rs1241709665: p.Ile20Phe, rs55648406: p.Arg49Gln) in the ET cohort (SKAT-O test p-value = 6.20e-08). *TUB* encodes a transcription factor predominantly expressed in neuronal cells and has been previously implicated in obesity. ChIP-seq analyses of the TUB transcription factor across different regions of the mouse brain revealed that TUB regulates the pathways responsible for neurotransmitter production as well thyroid hormone signaling. Together, these results support the association of rare variants in *TUB* with ET.

## Author summary

Essential tremor (ET) is the most common adult-onset movement disorder and in most affected families it appears to be inherited in an autosomal dominant pattern. The causes of essential tremor are unknown. Although many genetic studies in affected families and sporadic cases of ET have shown that genes may play a role, it has proven quite challenging to identify the specific genetic variants involved. Here, we use state-of-the-art technologies to identify the role of genetic variants on ET through exome sequencing of a large affected ET family and subsequent validation in a large population of cases and controls. We show that rare nonsynonymous variants of the *TUB* gene are significantly enriched in ET cases versus healthy controls. Further studies of biological pathways regulated by TUB in the mouse brain reveal key pathways related to ET. Our work expands our knowledge of the genetic basis of ET.

## Introduction

Essential tremor (ET) is the most common adult-onset movement disorder, and is characterized by the presence of kinetic tremor [1–3]. The primary phenotypic characteristic of ET is postural tremor of the arms, but other parts of the body may also be affected, including the head, legs, voice, jaw, and facial muscles [1–3]. ET may appear at any age, but is most common in the elderly [4, 5]; its prevalence is estimated to be as high as 4% in people over age 40, and 14% in people over 65 years of age [6, 7]. A family history of ET is a risk factor for the disorder [8] and correlates with an earlier age of onset [9]. Twin studies highlight the contribution of both genetic and environmental factors to disease risk [8, 10].

In presumably monogenic tremor, family studies are consistent with an autosomal dominant model of inheritance with high but not complete penetrance [11].

Especially in linkage studies, some family members have clearly transmitted the allele to offspring’s but do not express the disease, suggesting incomplete penetrance [11].

In these families, the phenotype is usually fully penetrant by the age of 65 years [8]. Several linkage studies have identified the genetic loci ETM1 on 3q13 (including *DRD3* gene), ETM2 on 2p24.1 and ETM3 on 6p23 for ET [1, 12–14]. However, most of causative mutations are still unidentified. Genome wide-association studies of sporadic cases of ET also identified multiple new risk loci (e.g. *SLC1A2* [15], *LINGO1* [16], *STK32B* [17], *PPARGC1A* [17], *CTNNA3*[17]).

Recently, a number of exome sequencing studies have identified a number of candidate genes (e.g. variants in *VPS35*, *DNAJC13*, *HTRA2*, *NOS3*, *KCNS2*, *HAPLN4*, *USP46*, *SCN4A*, *TENM4*, and *FUS*) in presumably monogenic ET, but all lack confirmation in independent studies [9, 18–21]. These genes also cover at best only a small fraction of the genetic basis of ET.

Here we report the results of an exome sequencing study in a large Caucasian family with ET (10 affected and 6 un-affected family members) and a follow-up study in a cohort of 820 unrelated individuals with ET and 630 controls. We find that a variant of the gene encoding the well-known transcription factor TUB, which has been previously implicated in obesity, is associated with familial ET. We further show that mutations of the *TUB* gene are enriched in a cohort of essential tremor patients. Finally, we identified key biological pathways that are regulated by TUB in the brain, indicating new biological roles for this factor and its involvement in human neurological disease.

## Results

### Exome sequencing in a multiplex ET family

**Fig 1:**
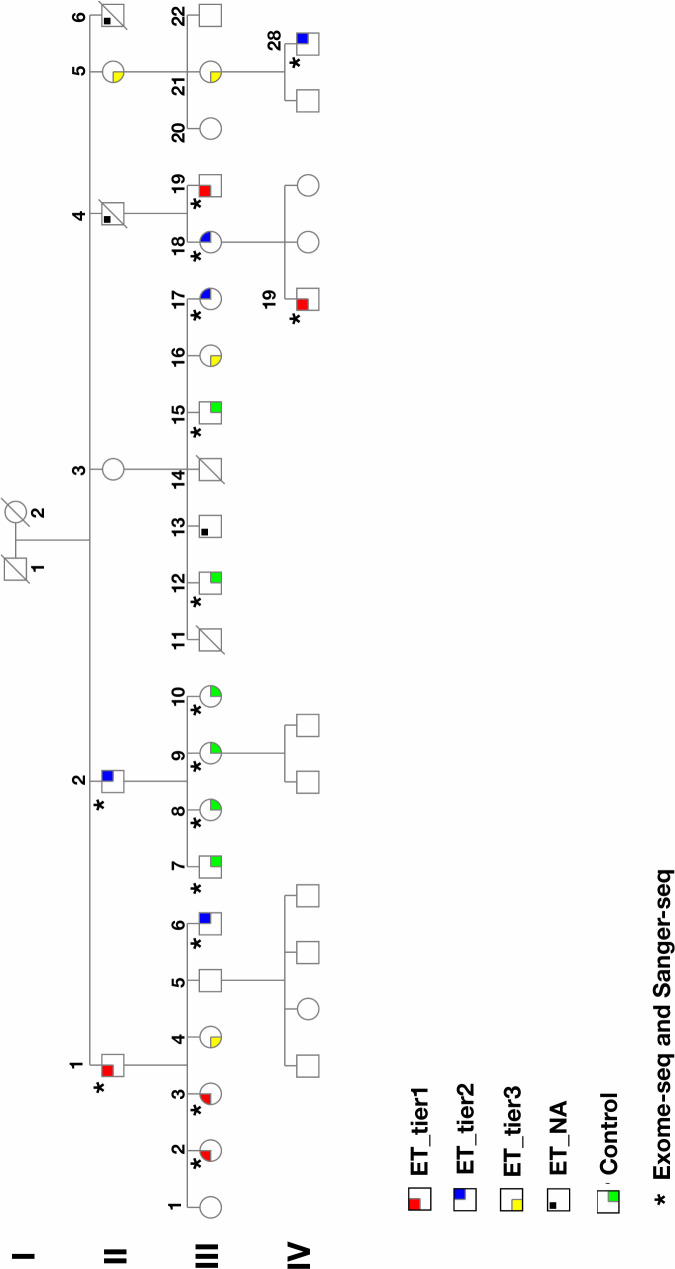
Pedigree of the multiplex ET family. Affected individuals are categorized in two different tiers. Tier 1, highlighted in red, are participants whom we are most certain have familial tremor (definite). They have all been diagnosed by a physician or neurologist and have clear symptoms of tremor that the age of onset was before their 65s. Tier 2, highlighted in blue, are participants who do state they have not been diagnosed with tremor by a physician, but describe clear symptoms of tremor in their questionnaires, with onset before their 65s. All have described the tremor interfering with their activities of daily living in some way. Individuals highlighted in yellow are Tier 3, whom the tremor is most unclear. Some are unsure if their symptoms are actually ET or not. The symptoms are generally very mild and, in many cases, intermittent. None have been diagnosed by a physician, and for some symptoms didn't onset until their 70s or later. Individuals highlighted in black are reported to have tremor, but these individuals are not participating. Controls, highlighted in green, are in their mid 50s or older and have no symptoms of tremor, according to their questionnaires. (Please note that this is a short version of the pedigree. (The full pedigree is shown in S1 Fig).

As described in methods, we developed a questionnaire method, which included a spiral-drawing test to screen a large family in which many members had symptoms of ET (Fig 1 and S1 Fig). Sixteen individuals were selected; ten were classified as ET cases in that they had tremor onset prior to age 65 and also failed the motor coordination test in the questionnaire (Please see S1 Text). Six were classified as unaffected controls in that they were over 60 years of age and had good motor coordination. It is of note that one individual from the cases had a history of thyroidectomy.

We performed exome sequencing (>50-fold average coverage) on all sixteen individuals (S2 Fig and S1 Table). We identified a total of 63,697 variants. We then excluded variants with a minor allele frequency (MAF) more than 0.02 in public databases (*e*.*g*. dbSNP138, 1000 Genome, ExAC) (Table 1). The reason for selecting the 0.02 MAF threshold is that ET is a relatively common condition (2.2% in general population, 4% in individuals aged over 40, and 14% in individuals aged over 60) [22].

**Table 1:** Variant filtering steps in multiplex ET family (10 cases and 6 controls).

Variant Filtering	ET family
Average Total Exonic Variants	62,460
1KG MAF < 0.02	13,127
UK 10K twin < 0.02	10,352
dbSNP 144 MAF < 0.02	10,209
NHLBI MAF < 0.02	9,397
EXaC MAF < 0.02	9,080
Missense variants	4,003
Stop gained/lost variants	165
Frameshift indels/variants	608
Present in at least 9 cases	6
Present in none of controls	1
Candidate	rs75594955

**Fig 2:**
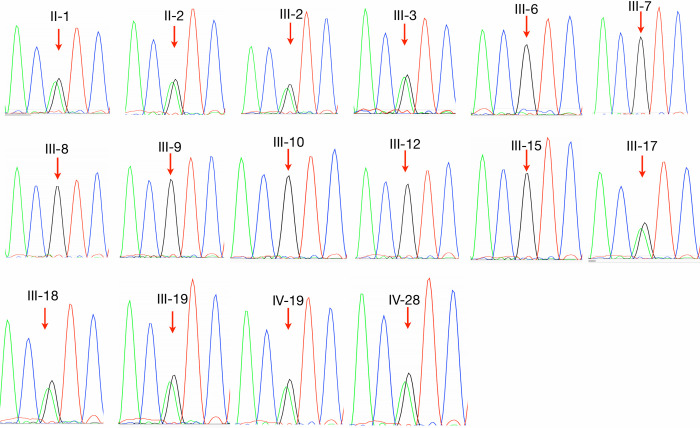
Sanger sequencing traces (ATG/ACT) showing the p.Val 431 Ile variant in exon 11 of the *TUB* gene (using sequencing primer 5`-AGCGAGTGGAAGAACAGCATTGCC-3`). The segregation of this variant has been confirmed in 15 available DNA samples (six controls and 9 affected individuals) from this family.

**Fig 3:**
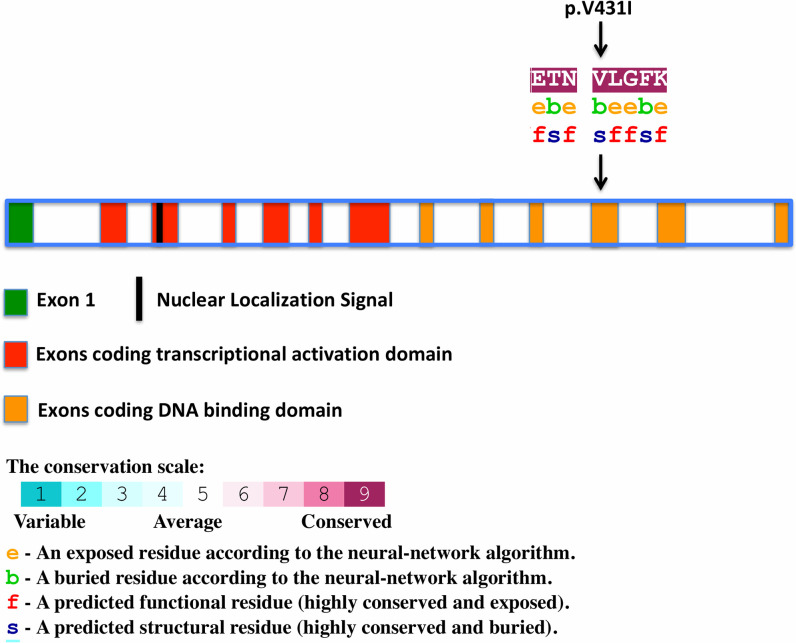
Schematic of the coding sequence of TUB gene and p.Val 431 Ile variant within TUB. The amino-acid sequence of TUB around (p.Val 431 Ile) colored according to the conservation scores. Conservation scores were calculated by ConSurf tool[49]. ConSurf estimates the evolutionary conservation of amino acid residues in a peptide based on the phylogenetic relations between homologous sequences as well as amino acid’s structural and functional importance.

We searched non-synonymous variants and splice sites as well as stop gain/loss variants in coding regions for those that were present in at least 9 out of 10 cases and none of the controls. These filtering steps resulted in the identification of a single non-synonymous heterozygous variant (rs75594955; NM_003320.4:c.1291G>A; p.Val431Ile) in exon 11 of the *TUB* gene (Table 1 and Table 2). The frequency of rs75594955 is less than 0.01 in most populations except the Finnish population, where it is 0.0114 (Exome Aggregation Consortium). The frequency of rs75594955 (NM_003320.4:c.1291G>A; p.Val431Ile) in the U.K. biobank is 0.0094 (https://www.ukbiobank.ac.uk).We further confirmed the variant by Sanger sequencing (Fig 2) using sequencing primer 5`-AGCGAGTGGAAGAACAGCATTGCC-3`and showed its segregation in the family (Fig 2). p.Val431Ile resides in the DNA binding domain of this transcription factor (Fig 3). p.Val431Ile is present in 9 out of 10 family members with ET. It is high likely that tremor in individual not carrying the TUB variant (from tier 2) is due to thyroidectomy and/or is a phenocopy. Since this individual has a history of thyroidectomy, it indicates that the genetic basis of his tremor is different from other affected members in the family, as tremor has been reported in patients with thyroidectomy [23]. We also genotyped (rs75594955; NM_003320.4:c.1291G>A) in Tier 3 individuals whom the tremor is most unclear. There are four individual who are in Tier 3 (Fig 1 and S2 Table), of which one individual has a diagnosis of ALS. Interestingly, all individuals in Tier 3 carry TUB mutation (S2 Table), except the individual with the diagnosis of ALS.

**Table 2:** Exome sequencing results in the multiplex ET family.

Chr.	Gene	Nucl. change	AA change	*dbNSFP	**CADD	PolyPhen	SIFT
11	TUB	c.1126G>A	p.Val431Ile	Deleterious	22.7	Possibly Damaging	Tolerated

* dbNSFP Functional Predictions and Scores 3.0, GHI [24]. Pathogenic computational verdict because 8 pathogenic predictions from DANN, GERP, dbNSFP.FATHMM, LRT, MetaLR, MetaSVM, MutationAssessor and MutationTaster (vs 1 benign prediction from PROVEAN).

** Combined Annotation Dependent Depletion.

If we relax the filtering criteria to accommodate variants present in fewer cases (*e*.*g*. present in at least six out of ten cases and none of controls), we find an additional variant; “rs141514527” in coding region of *ZC3H4* gene. However, this variant is considered benign based on dbNSFP functional predictions [24].

In addition, we analyzed IBS (identical by state) regions among cases and controls through beagle software v5.1. Overall, we identified 15,000 IBDs (identical by descent) segments, and the distribution by count (normalized based on chromosome size) is plotted in S3 Fig. However, we did not identify IBD segments that segregated with the disease, either near the *TUB* gene or elsewhere in the genome.

### Large-scale candidate gene resequencing results

To further test whether rare variants within *TUB* might be associated with ET, we examined their prevalence in a large cohort of ET cases relative to controls. We initially sequenced 1478 individuals, of whom 840 were affected by ET and 638 were unaffected. Deep sequencing was performed using a 21-amplicon PCR assay and the Fluidigm Access Array technology. A filter of >20 mean depth per sample was applied to call variants. We excluded samples with low read coverage, and after stringent quality control, the final dataset was comprised of 1450 individuals, including 820 disease cases and 630 controls. We identified 380 high quality variants of the *TUB* gene within the control group, of which 42 were predicted to be deleterious mutations. We also identified 487 variants of *TUB* in the ET group, of which 92 variants were predicted to be deleterious mutations according to the dbNSFP functional predictions and scores database [24] (Table 3). This represents a significant enrichment of predicted deleterious *TUB* mutations in the ET group compared to the control (Table 3). 159 individuals in the ET group (19.3%) carried predicted deleterious variants in *TUB*, whereas 55 individuals from the control group (8.7%) carried predicted deleterious variants within *TUB* (Fisher exact test statistic p-value = 5.30e-07; SKAT-O test p-value = 6.20e-08) (Table 3). The list of variants and their frequencies in cases and controls are shown in S1 Table. It is of note that the rs75594955 variant (p.Val431Ile) originally identified in the multiplex ET family is seen in 14 cases of sporadic ET cohort (820 cases) and six of controls (630 controls, consistent with 1% MAF in European population) (S3 Table). Moreover, we have identified additional rare nonsynonymous variants that are shown in S3 Table. For instance, the rs1241709665 variant (p.Ile20Phe) (MAF: 0.00006) is found in 27 cases of ET cohort and three controls. Additionally, the rs55648406 (p.Arg49Gln) (MAF: 0.003) and a new variant (chr11:8122535 (G/C)) are only seen in 12 cases of the ET cohort and none of the controls (S3 Table). Although the Fisher test does adjust for the sample imbalance in our case-control study (190 more cases have been sequenced than controls), we performed a bootstrap approach to be resampling the cases to match the number of controls (630 cases and 630 controls), 1000 times. Our analysis showed that the number of individuals, who carry at least one nonsynonymous mutation in *TUB*, ranges from 108 to 137 cases from these 630 cases (S4 Fig and S4 Table).

**Table 3:** *TUB* variants in the replication cohort of 1450 individuals.

	Control group (630 controls)	ET group (820 cases)
**Total Variants**	380	496
**1KG MAF < 0.02**	373	490
**EXaC MAF < 0.02**	371	485
**dbSNP 144 MAF < 0.02**	356	484
**NHLBI MAF < 0.02**	356	484
**UK 10K twin < 0.02**	356	484
**Missense variants**	77	103
Deleterious variants*	42	92
Heterozygote individuals**	55 out of 630	159 out of 820
The two-tailed P value***	5.30e-07	
**SKAT-O test p-value**	6.20e-08	

MAF, minor allele frequency; 1KG, 1000 Genome project phase 3; EXaC, Exome Aggregation Consortium version 0.3; dbSNP 144, Database of Single Nucleotide Polymorphism, NCBI; NHLBI, Exome Variant Server, NHLBI GO Exome Sequencing Project (ESP), UK10K, UK 10,000 project, ALSPAC—Variant Frequencies 2013-11-01, GHI.

* dbNSFP Functional Predictions and Scores 3.0, GHI [24].

**The number of individuals from the control and the tremor group who have a heterozygote mutation for damaging variants.

***Fisher exact test p-value.

In addition, as a control we performed an enrichment analysis for the rare but synonymous variants between cases and controls. This analysis provided a locally matched null distribution to control for potential differences in nonsynonymous variants between cases and controls. Our analysis showed no difference in enrichment of rare synonymous variants between cases and controls (P-value = 0.2, S5 Table). Therefore, we conclude that nonsynonymous variants in *TUB* are significantly enriched in ET case relative to controls.

### Identifying genome-wide DNA binding sites for TUB

**Fig 4:**
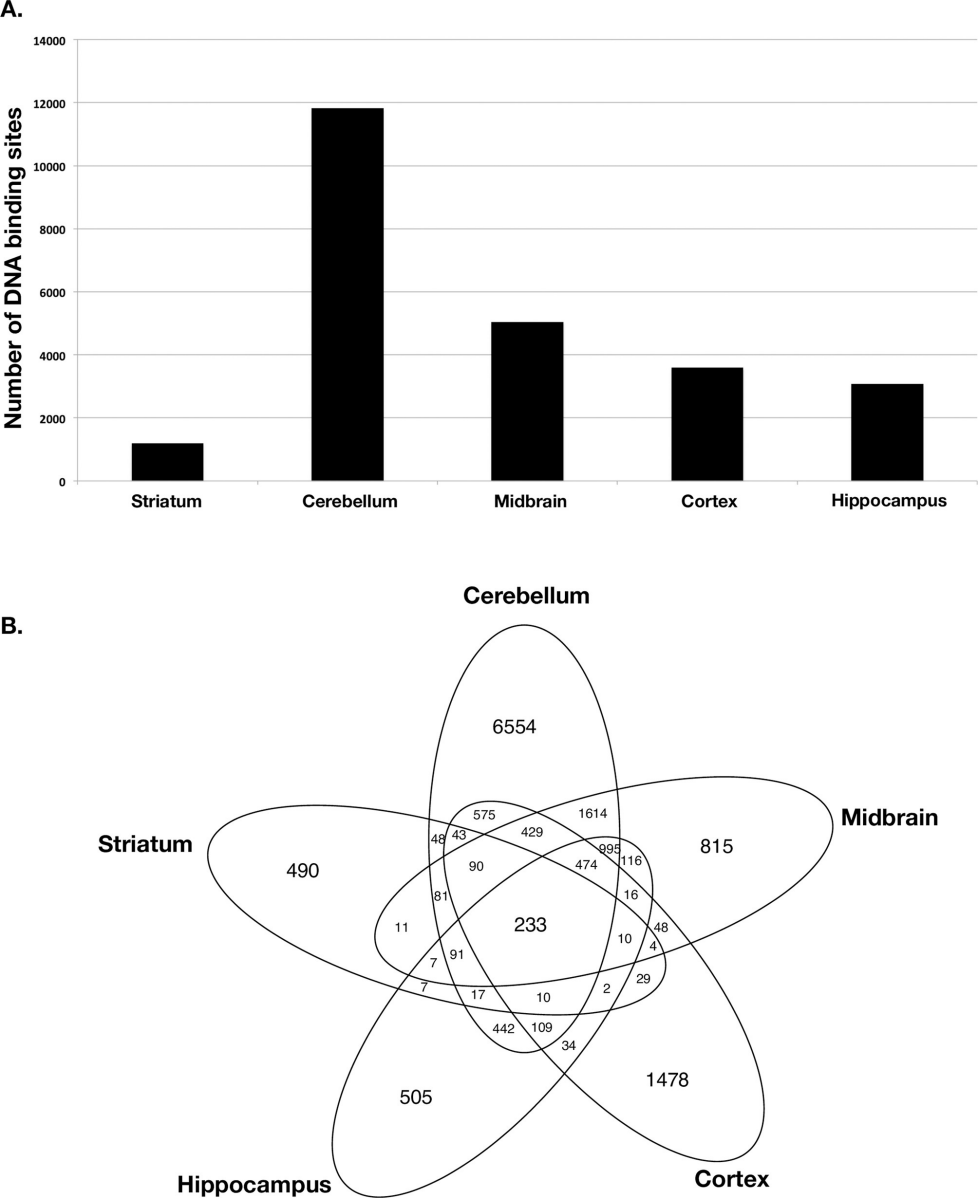
A) Number of TUB binding sites across different regions of the mouse brain by ChIP-seq. B) The overlap of TUB binding sites across different regions of the mouse brain.

**Fig 5:**
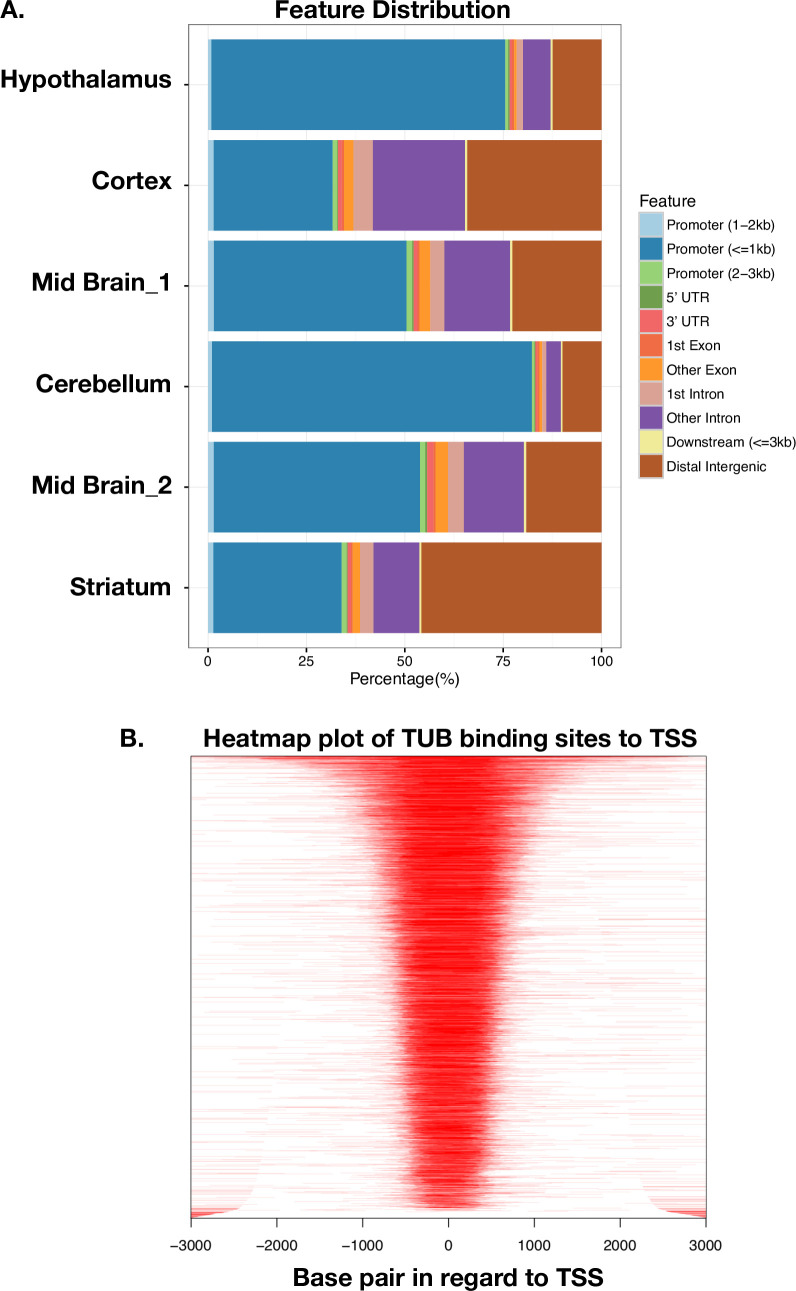
A. Genomic annotation of ChIP peaks across different regions of the mouse brain. B. Heatmap plot of ChIP binding sites to transcription start sites (TSS) regions.

**Fig 6:**
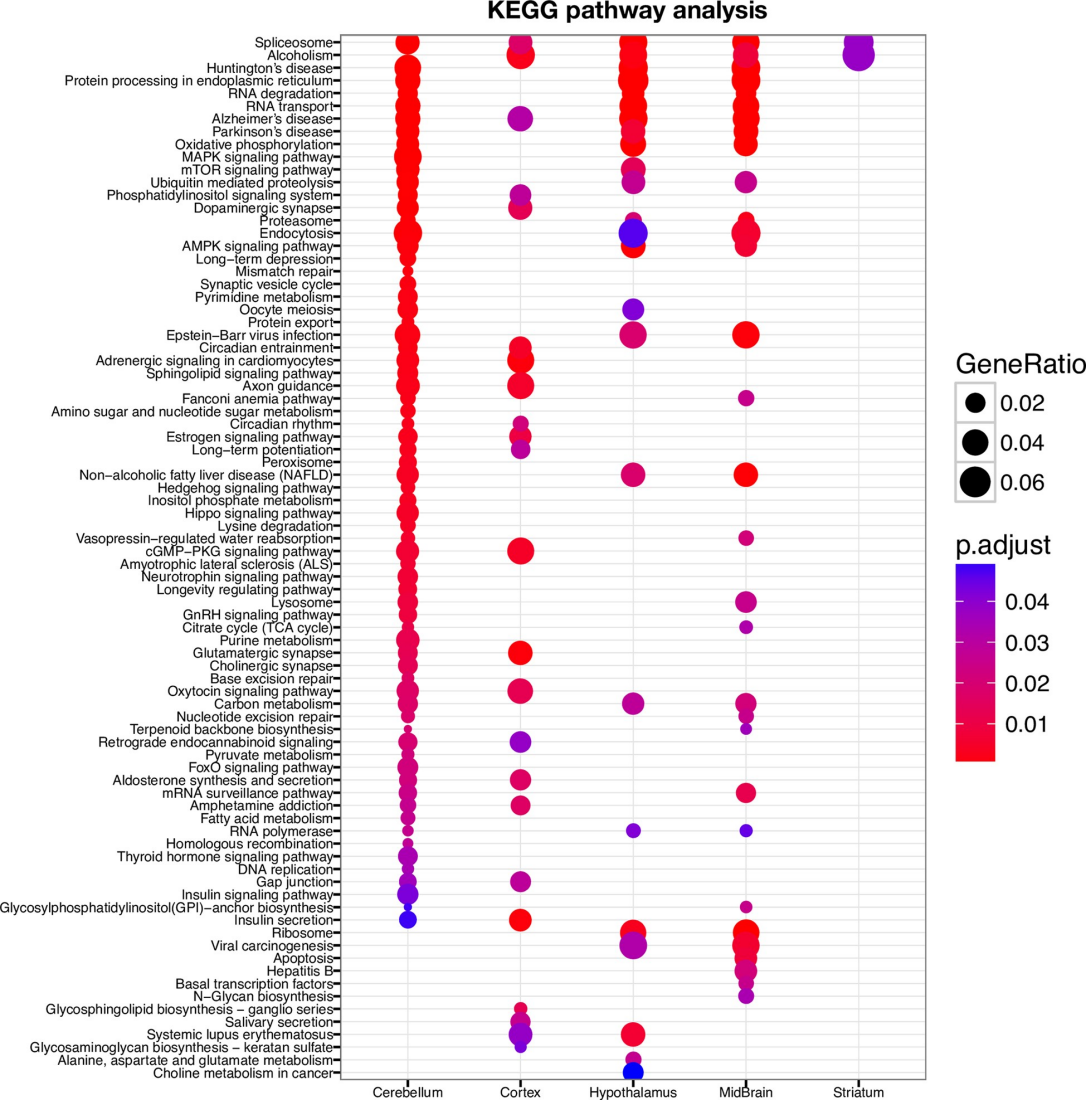
KEGG pathway enrichment analyses of TUB binding sites.

TUB is a member of the Tubby family of bipartite transcription factors [25] that are predominantly expressed in neuronal cells. TUB plays an important role in the maintenance and function of neurons during development and post-differentiation via thyroid hormone and G-protein-coupled receptor signaling [25–27]. In order to better understand how TUB functions, we identified DNA binding sites of TUB protein in the brain by ChIP-seq experiment. We used different parts of the mouse brain (cerebellum, striatum, midbrain, hypothalamus, and cortex). Histopathology studies have shown that ET is most likely a disease of the cerebellum [28]. Interestingly, our ChIP-seq data detected the highest number of TUB binding sites in the cerebellum (11,828 binding sites) (Fig 4 and S6 Table). We identified a smaller fraction of binding sites in cortex (3,596 binding sites), mid-brain (5,066 binding sites), striatum (1,194 binding sites), and hypothalamus (3,073 binding sites) (Fig 4). Furthermore, most of the TUB binding sites in the cerebellum fell within the promoter region, whereas for striatum, mid-brain and neocortex, most peaks fell within intragenic regions (Fig 5). The distribution of TUB binding sites across the genome is shown in S5 Fig. Interestingly, pathway enrichment analysis shows that TUB regulates a broad spectrum of pathways in the cerebellum. This includes key pathways related to neurotransmission, such as those involved in the dopaminergic and cholinergic synapses (S6 Fig) as well as axon guidance. Dopamine (DA) is an important slow neurotransmitter in the brain, where it controls a variety of vital functions including locomotor activity [29]. Additionally, acetylcholine (ACh) is a neurotransmitter in nervous system (CNS) that facilitates many functions, such as learning, memory, attention and motor control [30]. Intriguingly, the thyroid hormone signaling pathway is also under TUB regulation in the cerebellum (Fig 6), revealing a potential interplay between thyroid hormone and TUB. Moreover, across different regions of the brain, the cerebellum is the only region of the brain in which *TUB* expression is tightly regulated by thyroid hormone [27].

## Discussion

In the present study we reported the results of exome sequencing for a large ET family that led to the identification of a variant (p.Val431Ile in exon 11) in the *TUB* gene associated with ET. The frequency of rs75594955 is less than 0.01 in most populations except the Finnish population, where it is 0.01099. p.Val431Ile resides in the DNA binding domain of this transcription factor (Fig 2).

A follow-up study of 820 individuals with ET and 630 control individuals showed significant enrichment of nonsynonymous *TUB* variants in the ET cohort (SKAT-O test p-value = 6.20e-08). We have identified nonsynonymous rare variants (e.g. rs1241709665 (p.Ile20Phe: MAF: 0.00006384) and rs55648406 (p.Arg49Gln: (MAF: 0.003802)) in addition to rs75594955 (p.V431I). The rs1241709665 variant is found in 27 cases of ET cohort and three of controls, while the rs55648406 and a new variant (chr11:8122535 (G/C)) are only found in 12 cases of ET cohort and none of the controls (S3 Table).

*TUB* codes for the tubby bipartite transcription factor [25] and has been implicated in maturity-onset obesity, insulin resistance, retinal degeneration, and neurosensory hearing loss in mice, with *TUB* mutations associated with each of these phenotypes when inherited in an autosomal recessive mode. Moreover, it has been shown that *TUB* is predominantly expressed in neuronal cells and plays an important role in the maintenance and function of neurons during development and post-differentiation via thyroid hormone and G-protein-coupled receptor signaling [25–27]. Our ChIP-seq experiment across different regions of brain revealed that the TUB protein likely plays a greater role in neuronal regulation than previously thought, and identifies the cerebellum as a major target tissue region in the brain. Moreover, the TUB protein modulates a broad spectrum of pathways, including key pathways relevant for tremor phenotype such as those involved in dopaminergic and cholinergic synaptic transmission, as well as axonal guidance.

Dopamine (DA) is an important slow neurotransmitter in the brain, where it controls a variety of vital functions [31, 32]. The most important function of dopamine in the brain is regulation of locomotor activity [33]. Defects in this pathway are known causes of hyperkinetic as well as hypokinetic movement disorders [14, 34, 35]. Additionally, acetylcholine (ACh) is a neurotransmitter which facilitates many processes of the central nervous system, including learning, memory, attention and motor control [30]. Moreover, it has been shown that thyroid hormone plays a role in the release of both dopamine and choline neurotransmitters [36], and our data shows that TUB regulates thyroid hormone signaling in the cerebellum. It has been shown that thyroid hormones (T3 and T4) also positively regulate *TUB* gene expression in vivo and in cell culture [26]. Our data, in conjunction with literature, suggests a positive mutual interaction between *TUB* and thyroid hormone, particularly in the cerebellum.

*TUB* expression is lower in rats with hypothyroidism, and T3 treatment of cultured neuronal cells increased *TUB* expression significantly [26]. Intriguingly, *TUB* is one of the few genes under thyroid hormone regulation in the adult brain, whereas most thyroid target genes are only thyroid dependent in the early postnatal period [37]. Koritschoner et al (2001) has shown the effect of congenital hypothyroidism on *TUB* expression in the cerebellum [26]. Their results indicate that in animals born with congenital hypothyroidism, the initiation of T3/T4 treatment in adulthood induces a low level of *TUB* expression; however, this effect is seen only in the Purkinje cells of the cerebellum, and not in other regions of the brain [26].

Our pathway enrichment analysis data also show that TUB regulates thyroid hormone signaling only in the cerebellum and not other parts of the brain. Importantly, histopathologic studies have shown that ET is a disease of the cerebellum and seems to be centered on the Purkinje cells [38–42].

We note that our study did not find correlation with any the previously identified candidate genes for familial cases of ET. This highlights the great heterogeneity in the pathogenicity of ET and/or presence of different clinical subtypes for ET.

Moreover, additional research is required to evaluate the TUB variant in the pathogenicity of ET. Therefore, in the medical genetics setting this variant can be considered as variant of uncertain significance.

In conclusion, we report the co-segregation of a *TUB* variant with ET in a large multiplex family and additionally confirm this association in a replication cohort of 820 individuals with ET and 630 controls, supporting *TUB* variants as risk factors for familial ET. We also identified the dynamic of TUB binding sites in different regions of brain, and found key pathways potentially relevant to the ET phenotype.

## Material and methods

### Ethics statement

Human study: The institutional review board at Stanford University reviewed and approved this project, and all participants provided written informed consent.

Animal experiments: All animal procedures were approved by Stanford’s Administrative Panel on Laboratory Animal Care and were in accordance with institutional and national guidelines. All mice were housed in the Comparative Medicine Pavilion, and their care was monitored by the Veterinary Service Center at the Stanford University.

### Family description

The family pedigree is shown in Fig 1. Thirty family members provided saliva samples for DNA extraction. All participants completed a validated questionnaire (please see S1 Text) for evaluating tremor status. This also included a manual dexterity test to assess writing coordination. The study team, including a neurologist, who categorized participants as affected with ET and healthy controls, evaluated the questionnaires and tests. Whole exome sequencing was performed on 10 individuals who were either clearly affected with familial ET with onset prior to 65 years of age and six who were clearly unaffected in their 50s or older. Of the 10 family members who were affected by familial ET; five have been diagnosed for familial ET by their primary care physician or neurologist (Tier 1), and five have been diagnosed based on their questionnaire`s results and described clear symptoms with onset age before their 65 years (Tier 2) of age. All affected individuals have described that the tremor interfering with their activities of daily living. We excluded also one individual who reported symptoms of tremor but also carried a diagnosis of ALS. We also excluded tier 3 individuals whom the tremor is most unclear. Some are unsure if their symptoms are actually ET or not. The symptoms are generally very mild and, in many cases, intermittent. None have been diagnosed by a physician, and for some symptoms didn't onset until their 70s or later. We also excluded young healthy un-affected individuals (less than 50 years) as they may develop ET later in life. Finally, a total of 16 individuals remained of who 10 were classified as affected and 6 unaffected (Table 4).

**Table 4:** Clinical Status of Individuals in the Multiplex ET Family.

ID	Tremor Status	Group	Age	Age of onset	Thyroid status	TUB genotype
III-9	Un-affected	Control	65yo	NA	Normal	GG
III-7	Un-affected	Control	62yo	NA	Normal	GG
III-8	Un-affected	Control	60yo	NA	NA	GG
III-15	Un-affected	Control	71yo	NA	NA	GG
III-12	Un-affected	Control	78yo	NA	NA	GG
III-10	Un-affected	Control	68yo	NA	NA	GG
II-1	Affected	Tier 1	100yo	60yo	Hypothyroidism	GA
III-2	Affected	Tier 1	75yo	63yo	NA	GA
III-3	Affected	Tier 1	66yo	40yo	NA	GA
III-19	Affected	Tier 1	71yo	60yo	Hypothyroidism	GA
IV-19	Affected	Tier 1	48yo	18yo	NA	GA
III-17	Affected	Tier 2	69yo	55yo	NA	GA
II-2	Affected	Tier 2	93yo	50yo	NA	GA
III-6	Affected	Tier 2	64yo	36yo	Thyroidectomy	GG
III-18	Affected	Tier 2	79yo	60yo	Hypothyroidism	GA
IV-28	Affected	Tier 2	39yo	18yo	NA	GA

NA, not applied

### Exome sequencing and SNP calling

Exome capture and library preparation were performed using the Agilent SureSelectXT HumanAllExon V5 (product No. 5190–4631). Briefly, 3 μg of gDNA was sheared to a peak size of 150–200 bp using a standard Covaris manufactures protocol. Fragmented genomic DNA was purified using Agencourt AmpPure XP beads (Beckman Coulter) to remove fragments < 100 bp. Then, according to the manufacturer’s instructions, the purified DNA fragments were end-repaired, A-tailed and ligated to indexing-specific paired-end adaptors using the Agilent SureSelect Library Prep Kit, ILM. The adaptor-ligated libraries were amplified for five cycles with the SureSelect Primer and SureSelect Indexing Pre-Capture reverse primer. PCR reactions were then purified using Agencourt AMPure XP beads. To capture exonic regions, 500 ng of each prepared library was hybridized to biotinylated cRNA oligonucleotides for 24 hours at 65°C. The captured libraries were purified using Dynabeads MyOne Streptavidin T1 (Invitrogen). A post-capture PCR was then performed to amplify the captured libraries and to add the barcode sequences for multiplex sequencing for 14 cycles. Afterwards, amplified libraries were purified with AmpPure XP Beads. Qubit fluorometer and Bioanalyzer high sensitivity chips were used to determine the final concentration of each captured library. One library was prepared per sample. Libraries were pooled in groups of four, then paired-end sequenced using a single Illumina HiSeq 2000 lane at Macrogen. Raw fastq files were aligned to the human genome (hg19 version), with SNPs and indels called using the HugeSeq pipeline which utilizes published algorithms in a sequential manner (BWA for mapping the reads, SAMtools for detection of variants, Pindel for the detection of indels, and ANNOVAR for the annotation) [43].

### Exome sequencing Bioinformatics analysis

Since the inheritance pattern of familial ET in the family was consistent with autosomal dominance, we selected heterozygous exonic variants (excluding synonymous missense variants) with a minor allele frequency less than 0.02 in public database (1000 Genome project phase 3, Exome Aggregation Consortium version 0.3. dbSNP 144, Database of Single Nucleotide Polymorphism, NCBI. NHLBI, Exome Variant Server; NHLBI GO Exome Sequencing Project (ESP)). We also excluded variants that are located in repeat regions, segmental duplications or low complexity regions, as well as blacklisted genomic regions [44]. Finally, to identify ET-specific genetic variants from our exome sequencing data, we searched for non-synonymous SNVs, splicing sites as well as stop gain and loss variants in coding regions present in nine or more of ten patients with ET, but none of the controls. We did not require candidate variants to be present in all 10 affected individuals because ET is a common condition and a phenocopy might well be present in the family.

We have also checked the exome data for relatedness analysis. We used VCFtools option (vcftools—gzvcf file.vcf.gz—relatedness2). The kinship coefficients confirm the first-degree, second-degree and third degree relatives.

Moreover, we developed a pipeline to analysis IBD (Identity by descent) among cases and controls in the ET multiplexed family using VCF files. The VCF files were merged across samples and missing genotypes were imputed. We used beagle software v5.1 (ver. 24Aug19.3e8) to identify IBD segments.

IBDs were identified by refined-ibd (ver. 16May19.ad5) with option of "chrom = XX map = plink.chrXX.GRCh37.map window = 5.0 length = 0.5 lod = 2.0".

### Sporadic ET cohort

The sporadic ET cohort of 840 ET individuals was obtained from the Dept. of Neurology of Kiel University. ET was diagnosed as either “definite”,”probable”, or “possible” according to the clinical criteria proposed in the Consensus Statement on Tremor by the Movement Disorder Society [45]. All the 840 ET cases used in this study are in the “definite” category. The control group includes 638 individuals obtained from Universitätsklinikum Tübingen, Germany, and Stanford University.

### Candidate gene screening in ET cohort

840 individuals diagnosed with ET and 638 individuals as control were selected for *TUB* mutation screening. We coupled microfluidics-based multiplex PCR and deep sequencing (mmPCR-seq) to uniformly and simultaneously amplify the *TUB* coding sequence in 1478 samples. In brief, we designed 20 indexed primer pairs (Primer3web version 4.0.0) to cover all coding sequence of *TUB* gene (13 exons) (S7 Table). We then conducted 31 X 48 pools of 20 plex multiplex PCR primers to amplify *TUB* coding sequence in uniquely indexed 1478 individuals. The sizes of the amplicons range from 150 to 300 bp. We loaded DNA (50ng) and primer pools into the 48.48 Access Array IFC (Fluidigm) and performed targeted amplification according the manufacturer`s instructions. Individual PCR reactions took place in 35nl reaction chambers. PCR amplicons from a sample were pooled and barcoded by PCR (Fluidigm unidirectional sequencing protocol). All pools were combined at equal volumes and purified via AMpure XP beads. The library was sequenced using Illumina HiSeq with 101 bp paired-end reads. Paired-end reads were de-multiplexed and mapped onto the *TUB* coding sequence through a modified version of the Hugeseq pipeline [43].

### Large-scale candidate gene re-sequencing significance analysis

We test for the enrichment of rare nonsynonymous variants within TUB between cases and controls using a two-tailed fisher exact test as well SKAT-O (SNP-Set Kernel Association) test using SKAT test package in R [46, 47]. SKAT is an improved version of Burden test. While Burden test sums the minor allele counts in a given region to compare rare and common variants, SKAT sums the squares of score statistics given a region to further contrast rare and common variants. SKAT-O optimizes generalized sequence kernel association test (Generalized SKAT) over score statistics between 0 and 1 from N tests [46, 47]. Here we used 'linear.weighted' kernel for p-values of all tests. We have incorporated gender and age as covariates.

All samples used in this study are of Caucasian origin.

We also used custom scripts in R to perform a bootstrap approach to randomly selecting 630 cases (from our pool of 820 cases) to match the number of controls (630 controls).

### TUB ChIP-seq experiment

#### Collection of Mouse Brain Tissue

C57BL/6 mice were obtained from The Jackson Laboratory and housed in cages in temperature- and light-controlled environments with *ad libitum* access to food and water. Tissue from the following brain regions was collected from 12-week-old littermates: cerebellum, striatum, midbrain, hypothalamus and neocortex.

### Brain Tissue ChIP-seq procedure

Frozen mouse cerebellum, neocortex, mid-brain, striatum, and hypothalamus (10mg) tissue was crushed in the presence of liquid nitrogen in a sterile container, then re-suspended in 10 mL of cold PBS buffer on ice. The samples were crosslinked using 1mL 11X formaldehyde crosslinking solution (1% formaldehyde, 1 mM EDTA, 0.5 mM EGTA, 50 mM HEPES, pH 8.0) for 15 min at room temperature. Crosslinking was stopped by adding 733uL of 2M Glycine and incubating on a shaking incubator for 5 min at room temperature. Homogenates were centrifugated at 3000 g for 5 min at 4°C and washed with ice-cold PBS containing 1 μg/μl protease inhibitor cocktail (Sigma, P8340) and transferred to a 15mL glass Dounce homogenizer. The samples were homogenized first with loose pestle (10 times) and then tight pestle (15 times), and subsequently transferred to a new 15 mL falcon tube and centrifuged at 2500g for 3 min at 4C. Nuclear lysates were sonicated using a Branson 250 Sonifier (power setting 7, 100% duty cycle for 20s intervals), such that the chromatin fragments ranged from 50 to 2,000 bp. We used Tub (T-19): sc-1960 antibody (Santa Cruz Biotechnology, Inc.) for immuneparticipation (2 μg per 1 ml of cell lysate), according to the manufacturer instructions. Protein–DNA–TUB antibody complexes were captured on Protein A/G agarose beads (Millipore 16-156/16-266) and eluted in 1% SDS TE buffer at 65C. After cross-link reversal and DNA purification, the ChIP DNA sequencing libraries were prepared by NEBNext Ultra DNA Library Prep Kit for Illumina (Catalogue number NEB E7370L). Libraries were then pooled and sequenced using an Illumina HiSeq 4000.

#### Analysis of ChIP-seq Intensity

ChIP-seq Fastq data were mapped to the hg19 genome using bowtie version 1.1.1. with settings “bowtie–q–phred33-quals–X 2000 –fr–p 9 –S–chunkmbs 400”. *TUB* binding regions were identified from the aligned sorted bam files using MACS2 v. 2.1.0 with options “macs2 callpeak–bdg–t–g hs” [48]. Fold enrichment values were calculated using ChIP intensity measurements. We used CEAS^46^ and ChIPseeker^47^ for annotating ChIP peaks, and clusterProfiler^48^ for the enrichment analysis of gene clusters.

## Supporting information

S1 Fig The full pedigree of multiplex ET family. (TIF)

S2 Fig The exome coverage of the coding regions of genome (hg19) across samples. The plot shows the fraction of on-target coverage (Y-axis) and the read depth (X-axis) for the coding regions.
(TIF)

S3 Fig IBD pipeline and segments distribution across the genome. (TIF)

S4 Fig Distribution of number of cases with rare nonsynonymous mutations in TUB in randomly selecting 630 cases out of a pool of 820 cases, 1000 times. (TIF)

S5 Fig The distribution of TUB binding sites across the genome by CEAS program^46^ in A. Cerebellum, B. Striatum, C. Midbrain, D. Hippocampus and E. Cortex. The blue bars show the percentages of the mappable regions in the genome background and the red bars represent the percentages of the whole ChIP. P-values for the significance of the relative enrichment of ChIP regions with respect to the gnome background are shown in parentheses.
(TIF)

S6 Fig Representation of dopaminergic and acetylcholine pathways regulated by TUB. (TIF)

S1 Table Exome coverage statistics. (DOCX)

S2 Table Sanger sequencing results of rs75594955 in Tier 3 individuals. (DOCX)

S3 Table List of TUB nonsynonymous rare variants and their frequencies in sporadic cases of ET and controls. (XLSX)

S4 Table Number of cases with TUB mutation in resampling of cases to match controls. (XLSX)

S5 Table Rare and synonymous TUB variants in the replication cohort of 1450 individuals. (DOCX)

S6 Table List of TUB binding sites across different regions of the mouse brain (Striatum, Cerebellum, Midbrain, Cortex, Hippocampus). (XLSX)

S7 Table List of 20 indexed primer pairs used for re-sequencing of TUB gene in sporadic cases of ET and controls. (XLS)

S1 Text The questionnaire that includes a spiral-drawing test to screen the large multiplex ET family. (PDF)
