# Supplementary figures and images for: Candidate variants in TUB are associated with familial tremor
Source: PLoS Genet. 2020 Sep 21;16(9):e1009010. doi: 10.1371/journal.pgen.1009010 (PMC7529431; doi:10.1371/journal.pgen.1009010)

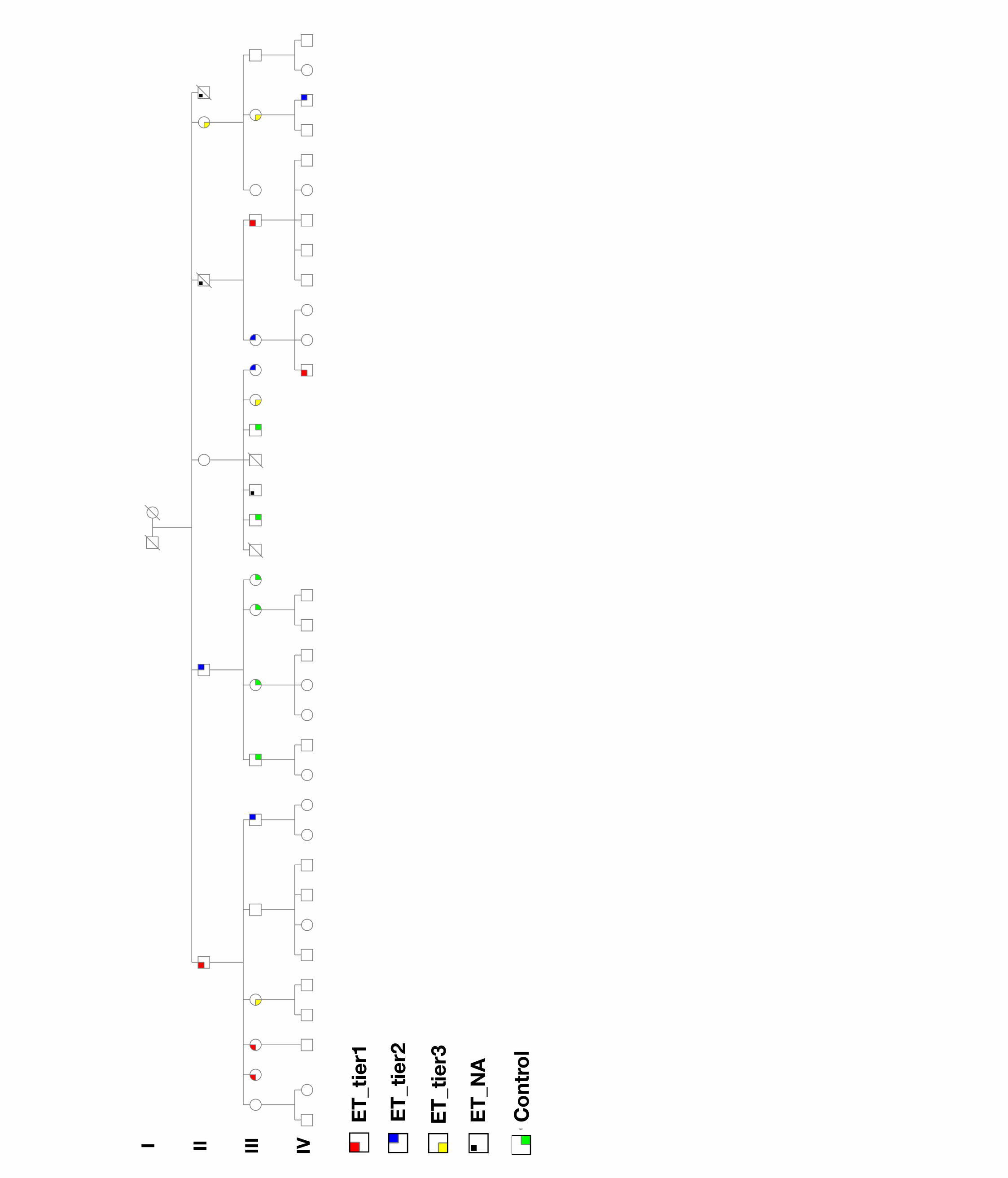

Supplement: S1 Fig — (TIF) [file pgen.1009010.s001.tif]

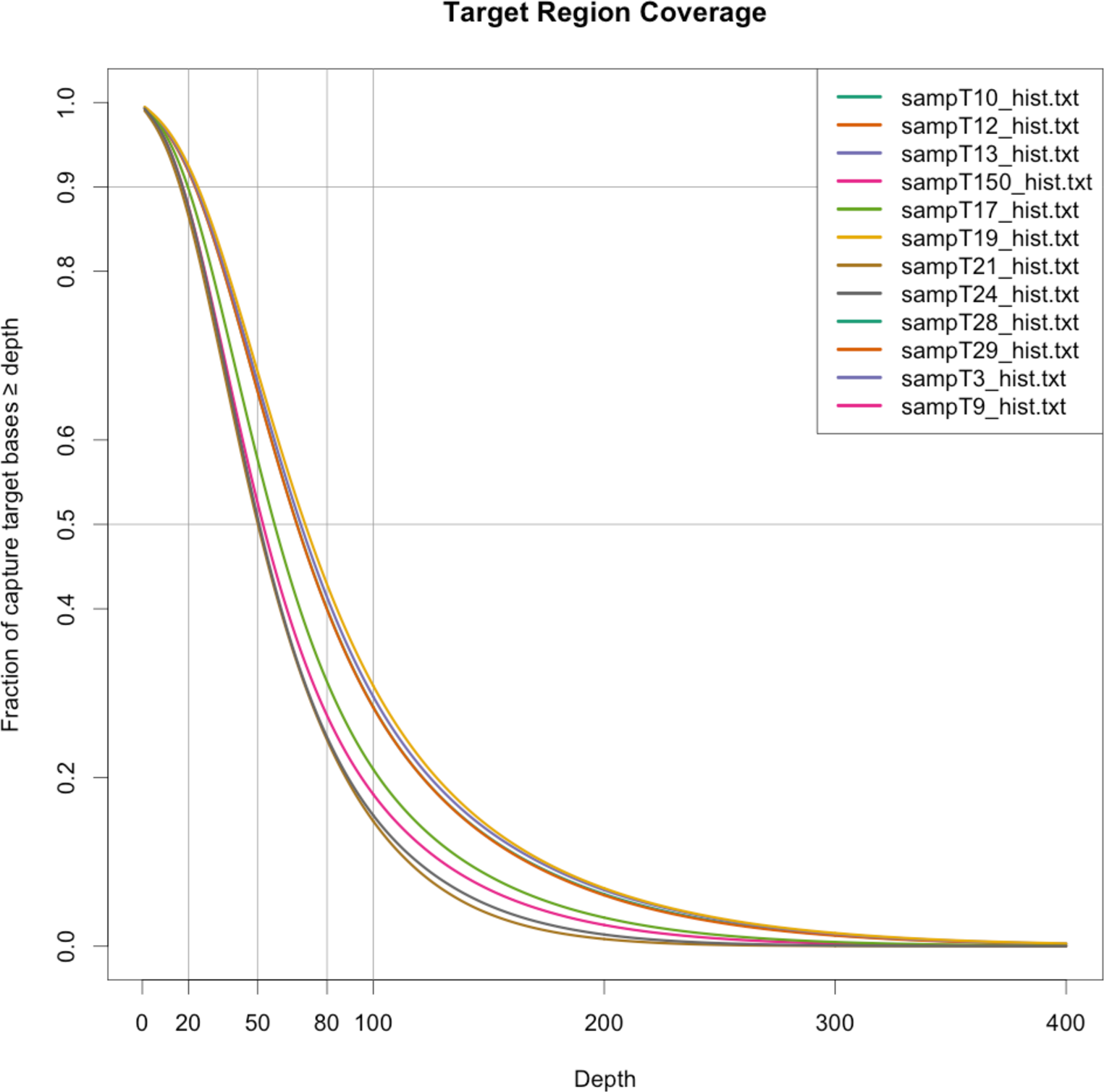

Supplement: S2 Fig — The plot shows the fraction of on-target coverage (Y-axis) and the read depth (X-axis) for the coding regions. (TIF) [file pgen.1009010.s002.tif]

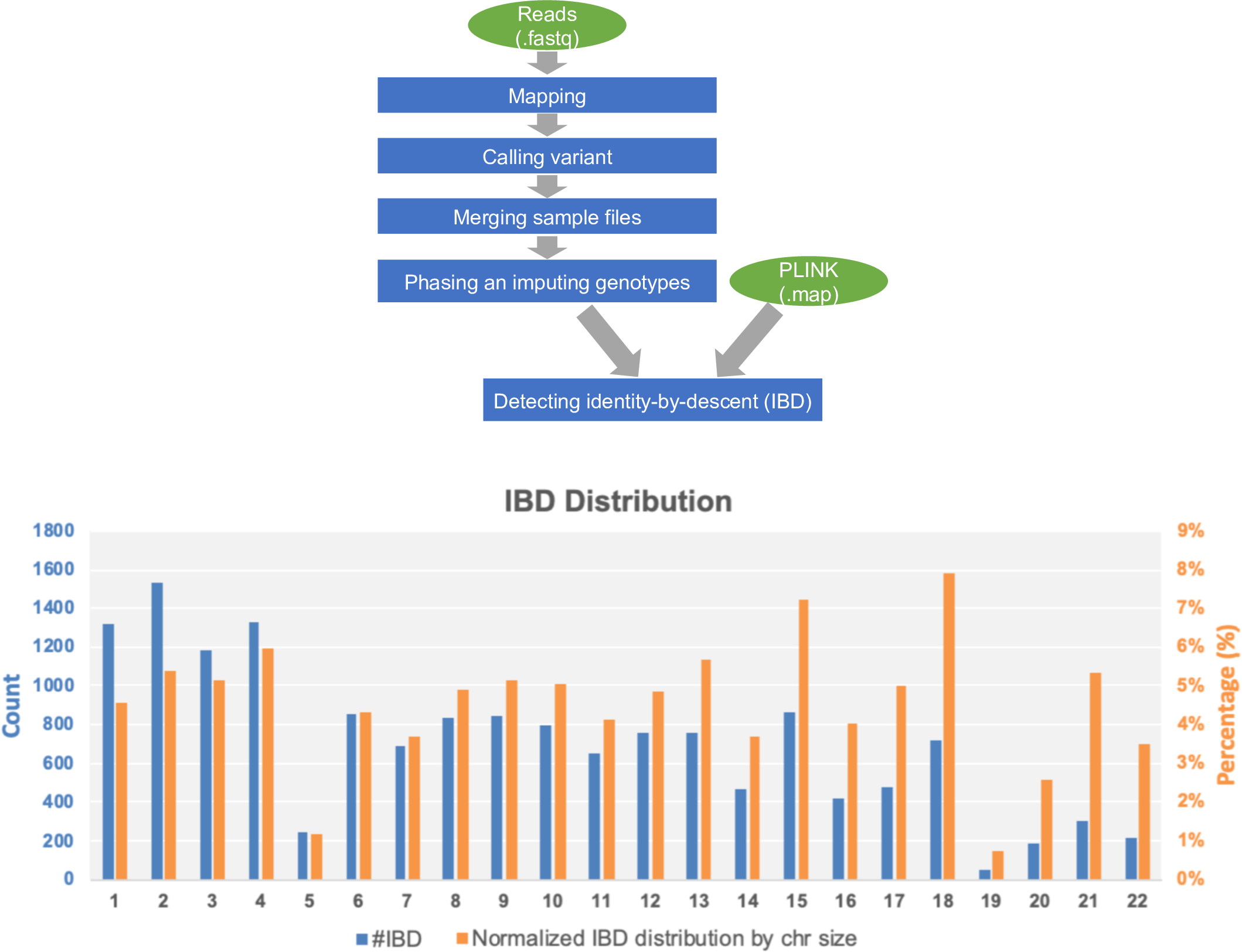

Supplement: S3 Fig — (TIF) [file pgen.1009010.s003.tif]

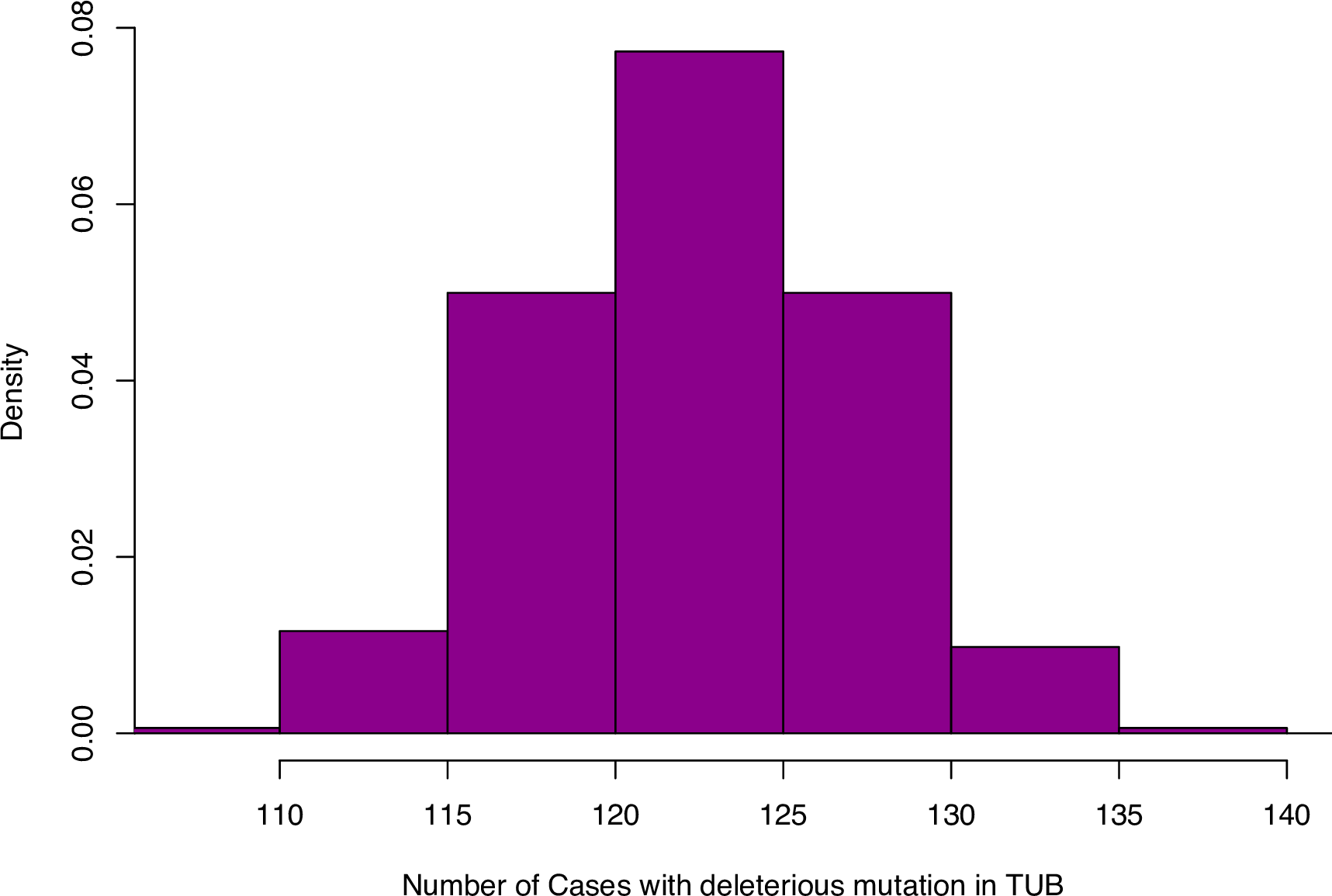

Supplement: S4 Fig — (TIF) [file pgen.1009010.s004.tif]

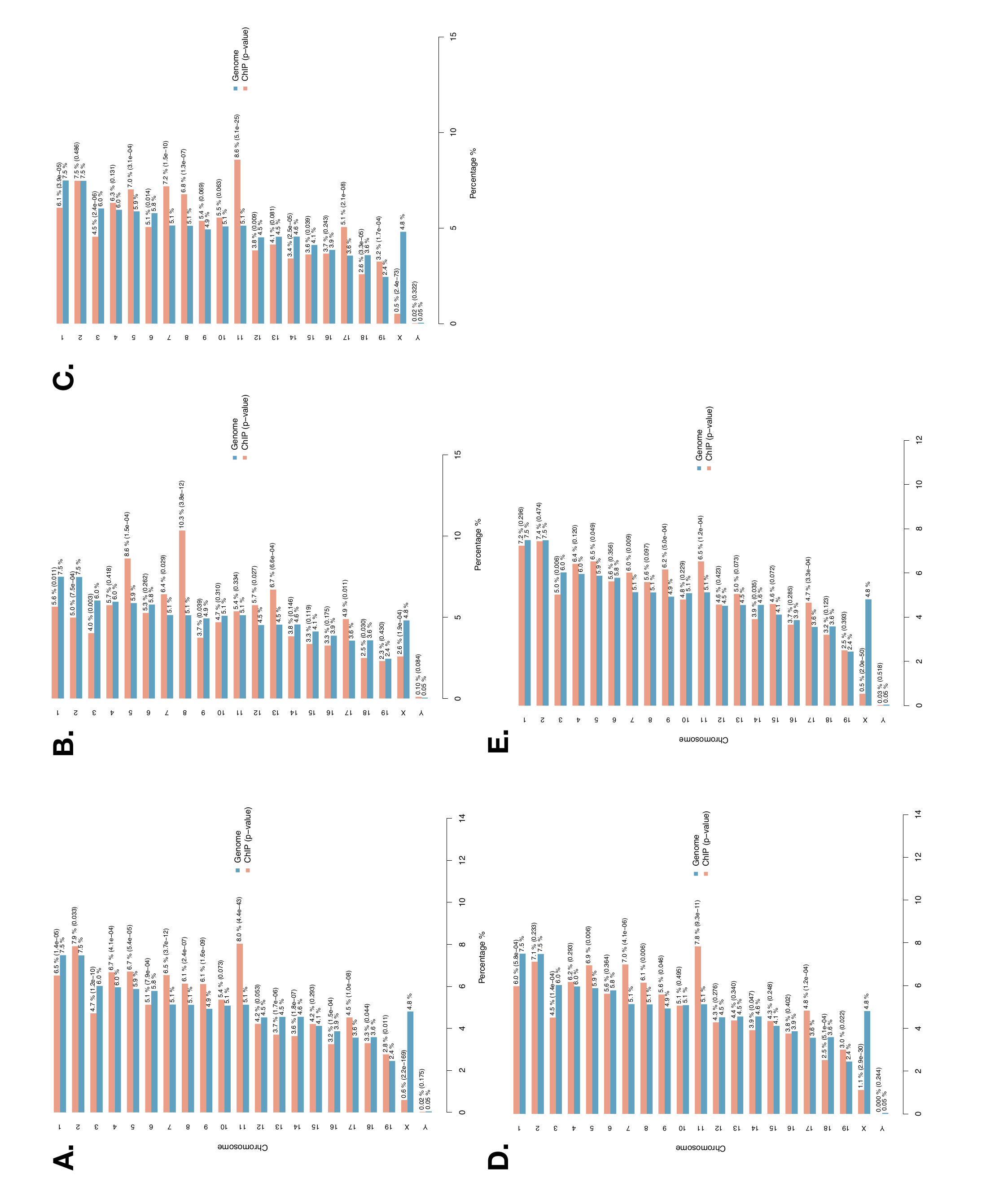

Supplement: S5 Fig — The distribution of TUB binding sites across the genome by CEAS program46 in A. Cerebellum, B. Striatum, C. Midbrain, D. Hippocampus and E. Cortex. The blue bars show the percentages of the mappable regions in the genome background and the red bars represent the percentages of the whole ChIP. P-values for the significance of the relative enrichment of ChIP regions with respect to the gnome background are shown in parentheses. (TIF) [file pgen.1009010.s005.tif]

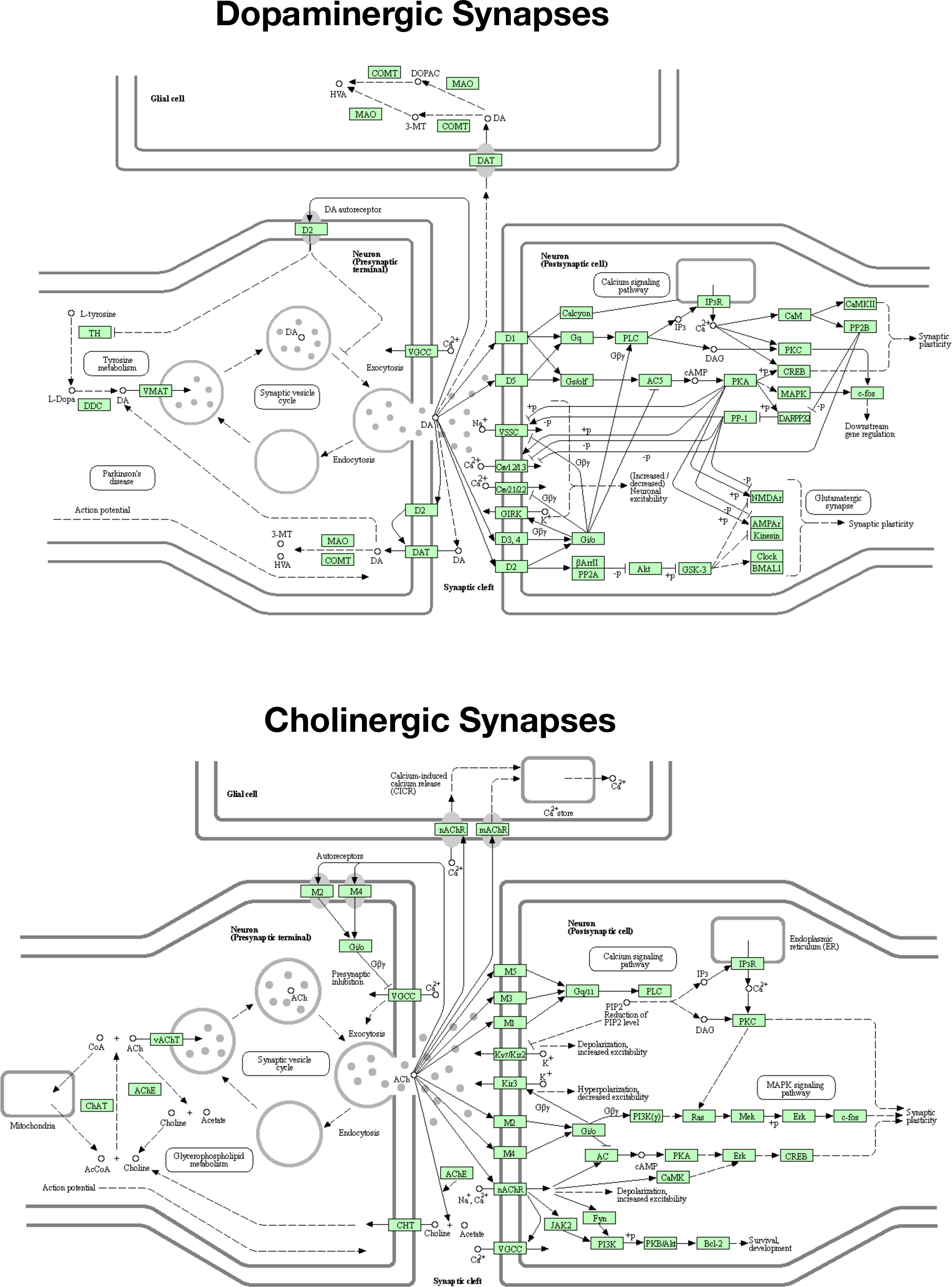

Supplement: S6 Fig — (TIF) [file pgen.1009010.s006.tif]
