# Supplementary material for: Candidate variants in TUB are associated with familial tremor
Source: PLoS Genet. 2020 Sep 21;16(9):e1009010. doi: 10.1371/journal.pgen.1009010 (PMC7529431; doi:10.1371/journal.pgen.1009010)
Supplement: S1 Table — (DOCX) [file pgen.1009010.s007.docx]

**S1 Table. Exome sequencing coverage data.**

| Sample | Total sequences | Read length (bp) | Percentage aligned reads (%) | Percentage read duplicates | Average on-target read coverage |
| --- | --- | --- | --- | --- | --- |
| II-1 | 59,914,973 | 101 | 94.80% | 27.60% | 58 |
| II-2 | 59,61,285 | 101 | 99.50% | 12.70% | 58 |
| III-2 | 83,959,288 | 101 | 99.80% | 17.60% | 70 |
| III-3 | 58,299,898 | 101 | 93.40% | 18.40% | 58 |
| III-6 | 86319300 | 101 | 99.70% | 18.10% | 73 |
| III-7 | 85,321,506 | 101 | 99.80% | 15.40% | 70 |
| III-8 | 82,488,860 | 101 | 99.70% | 16.50% | 66 |
| III-9 | 73,340,231 | 101 | 99.70% | 22.10% | 65 |
| III-10 | 65,482,280 | 101 | 99.60% | 11.90% | 58 |
| III-12 | 86,687,479 | 101 | 99.50% | 26.10% | 73 |
| III-15 | 82,488,860 | 101 | 99.70% | 16.50% | 66 |
| III-17 | 56,631,535 | 101 | 99.50% | 8.70% | 50 |
| III-18 | 65,236,686 | 101 | 99.60% | 14.80% | 72 |
| III-9 | 81,711,344 | 101 | 99% | 28% | 65 |
| IV-19 | 56,720,958 | 101 | 99.50% | 10.90% | 50 |
| IV-28 | 86,310,041 | 101 | 99.70% | 20.30% | 72 |
