# Supplementary material for: Candidate variants in TUB are associated with familial tremor
Source: PLoS Genet. 2020 Sep 21;16(9):e1009010. doi: 10.1371/journal.pgen.1009010 (PMC7529431; doi:10.1371/journal.pgen.1009010)
Supplement: S2 Table — (DOCX) [file pgen.1009010.s008.docx]

**S2 Table:** Clinical Status of Individuals in the Multiplex ET Family at Tier 3.

| **ID** | **Tremor Status** | **Group** | **Age** | **Age of onset** | **Thyroid status** | **TUB genotype** |
| --- | --- | --- | --- | --- | --- | --- |
| II-5 | Affected | Tier 3 | 91yo | 70yo | Hypothyroidism | GA |
| III-21 | Affected | Tier 3 | 67yo | 60yo | NA | GA |
| III-16* | Affected | Tier 3 | 71yo | 63yo | NA | GG |
| III-4 | Affected | Tier 3 | 69yo | 58yo | Hypothyroidism | GA |

*III-16 is affected by ALS disease and does not carry TUB mutation.
