# Supplementary material for: Candidate variants in TUB are associated with familial tremor
Source: PLoS Genet. 2020 Sep 21;16(9):e1009010. doi: 10.1371/journal.pgen.1009010 (PMC7529431; doi:10.1371/journal.pgen.1009010)
Supplement: S5 Table — (DOCX) [file pgen.1009010.s011.docx]

**S5 Table**. Synonymous *TUB* variants in the replication cohort of 1450 individuals.

|  | Control group  (630 controls) | ET group  (820 cases) |
| --- | --- | --- |
| **Total Variants** | 380 | 496 |
| **1KG MAF < 0.02** | 373 | 490 |
| **EXaC MAF < 0.02** | 371 | 485 |
| **dbSNP 144 MAF < 0.02** | 356 | 484 |
| **NHLBI MAF < 0.02** | 356 | 484 |
| **UK 10K twin < 0.02** | 356 | 484 |
| **Rare synonymous variants** | 27 | 36 |
| **Heterozygote individuals**** | 29 out of 630 | 49 out of 820 |
| **The two-tailed P value***** | 0.2 | |
| **SKAT-O test p-value** | 0.11 | |

MAF, minor allele frequency; 1KG, 1000 Genome project phase 3; EXaC, Exome Aggregation Consortium version 0.3; dbSNP 144, Database of Single Nucleotide Polymorphism, NCBI; NHLBI, Exome Variant Server, NHLBI GO Exome Sequencing Project (ESP), UK10K, UK 10,000 project, ALSPAC - Variant Frequencies 2013-11-01, GHI.

* dbNSFP Functional Predictions and Scores 3.0, GHI (PMID:26555599). **The number of individuals from the control and the tremor group who have a heterozygote mutation for synonymous variants. ***Fisher exact test p-value.
