# Supplementary material for: Candidate variants in TUB are associated with familial tremor
Source: PLoS Genet. 2020 Sep 21;16(9):e1009010. doi: 10.1371/journal.pgen.1009010 (PMC7529431; doi:10.1371/journal.pgen.1009010)
Supplement: S1 Text — (PDF) [file pgen.1009010.s014.pdf]

Familial Tremor Rating Scale

Person's Name: ___________________________ Date: ____ / ____ / ____

Gender: ☐ Male ☐ Female Date of Birth: ____ / ____ / ____

Have you ever been diagnosed for familial tremor?

- ☐ **Yes**
- ☐ **No, no diagnosis yet**

If YES:

At what age? __________________________

What type of tremor do you have according to your diagnosis? __________________________

Who did the diagnosis:

- ☐ **Primary Care Physician**
- ☐ **Neurologist**
- ☐ **Other Physician**

Where is the location of your tremor?

- ☐ **Hands**
- ☐ **Head**
- ☐ **Legs**
- ☐ **Other body parts**
- ☐ **I do not have tremor**

Tremor duration in years: __________________________

When was the first time that you noticed you had a tremor (in years)? __________________________

Does an alcohol drink improve your tremor?

- ☐ **Yes**
- ☐ **No**
- ☐ **Unknown**

Do you have a family history for tremor?

- ☐ **Yes**
- ☐ **No**
- ☐ **Unknown**

In case of family history, which family members are/were affected:

- ☐ **father**
- ☐ **mother**
- ☐ **brother**
- ☐ **sister**
- ☐ **children**
- ☐ **grandparents (paternal)**
- ☐ **grandparents (maternal)**
- ☐ **other**

What is the number of family members with tremor (excluding yourself)? _______________________

Where is your place of birth? City: ____________ State: _____________

Which ethnicity do you belong to?

- ☐ **African-American**
- ☐ **Asian**
- ☐ **Caucasian**
- ☐ **Hispanic**
- ☐ **Others. Please mention _______**

Have you ever taken any medications for controlling tremor?

- ☐ **Yes**
- ☐ **No**
- ☐ **Unknown**

Which medication(s) did you take? ____________

What was the effect of the medication(s)?

- ☐ **Effective**
- ☐ **Noneffective**

Do you have any other diseases?

- ☐ **Yes**
- ☐ **No**
- ☐ **Unknown**

If YES, please list them.

1- ………….

2- ………….

3- ………….

4- ………….

5- ………….

6- ………….

7- ………….

8- ………….

Do you take any other medications not related to tremor treatment?

- ☐ **Yes**
- ☐ **No**
- ☐ **Unknown**

If YES, please list them.

1- ………….

2- ………….

3- ………….

4- ………….

5- ………….

6- ………….

7- ………….

8- ………….

Handwriting:

____

This is a sample of my best handwriting (with the preferred hand)

Drawing: with □ right hand □ left hand

Join both points of the various drawings without crossing the lines.

Test each hand, beginning with the lesser, without leaning the hand or the arm on the table.

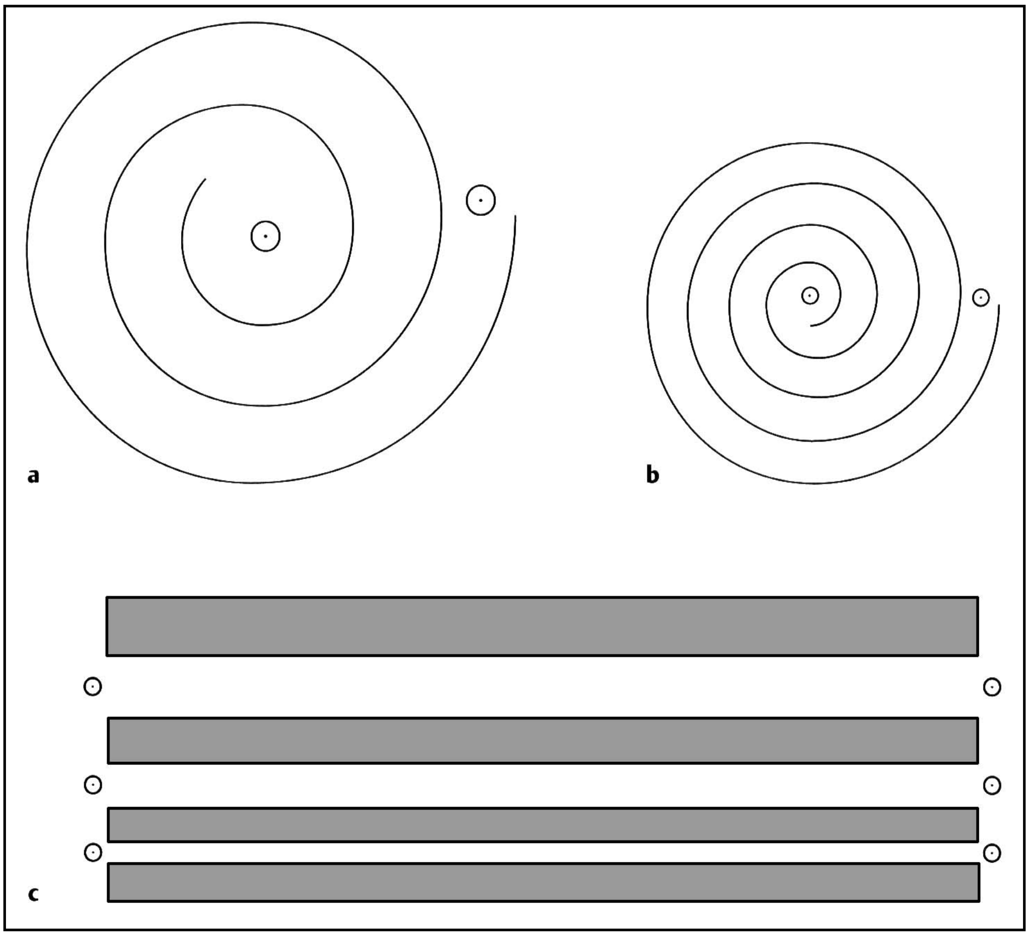

Drawing: with □ right hand □ left hand

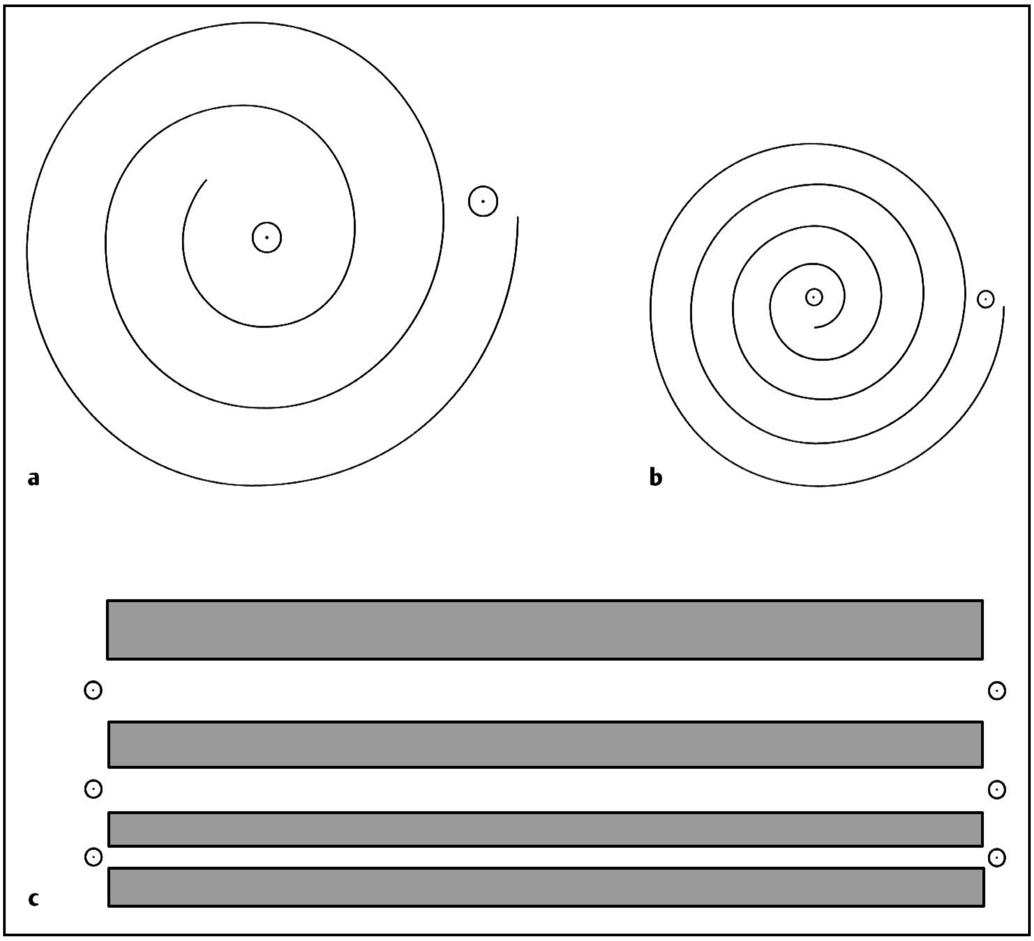

If you have tremor, did you take tremor related medications before doing drawing test?

- □ **Yes**
- □ **No**
- □ **Unknown**

If YES, please list them.

1- …………..

2- …………..

3- …………..

4- …………..

Quality of Life in Essential Tremor Questionnaire (QUEST)

Health Status

In general, how would you rate your overall health? (0=very poor health, 100=excellent/perfect health)
Circle: 0 5 10 15 20 25 30 35 40 45 50 55 60 65 70 75 80 85 90 95 100

Overall Quality of Life

Overall, how would you rate your quality of life? (0=very poor health, 100=excellent/perfect health)
Circle: 0 5 10 15 20 25 30 35 40 45 50 55 60 65 70 75 80 85 90 95 100

General Information

In the past month, has your tremor interfered with your sexual satisfaction? ☐ Yes ☐ No

In the past month, have you had side effects from tremor medications? ☐ Yes ☐ No

In the past month, have you been satisfied with the tremor control achieved by your medications? ☐ Yes ☐ No

Which most appropriately describes your work status?

- ☐ Never worked
- ☐ Not working, retired because of tremor
- ☐ Not working, retired NOT due to tremor
- ☐ Working full time
- ☐ Working part time

TREMOR SELF ASSESSMENT

For the purposes of this questionnaire, tremor is defined as uncontrollable shaking or quivering of the body part in question.

On a typical day, how many of your waking hours do you have tremor in ANY body part?
Circle: 0 1 2 3 4 5 6 7 8 9 10 11 12 13 14 15 16 17 18 19 20 21 22 23 24

Put a mark in the box to rate the severity of your tremor in each of the body parts listed below.

None - no tremor at any time
Mild - mild tremor not causing difficulty in performing any activities
Moderate - tremor causes difficulty in performing **some** activities
Marked - tremor causes difficulty in performing **most** or **all** activities
Severe - tremor **prevents** performing some activities

	None	Mild	Moderate	Marked	Severe
Head	☐	☐	☐	☐	☐
Voice	☐	☐	☐	☐	☐
Right arm/hand	☐	☐	☐	☐	☐
Left arm/hand	☐	☐	☐	☐	☐
Right leg/foot	☐	☐	☐	☐	☐
Left leg/foot	☐	☐	☐	☐	☐

For each question below, please mark the box which best describes your current situation.

For example: N R ~~S~~ F A

N = **N**ever/No
R = **R**arely
S = **S**ometimes
F = **F**requently
A = **A**lways/Yes
NA = **N**ot **A**pplicable

My tremor interferes with my ability to communicate with others.		N R S F A
My tremor interferes with my ability to maintain conversations with others.		N R S F A
It is difficult for others to understand my speech because of my tremor.		N R S F A
My tremor interferes with my job or profession.	NA	N R S F A
I have had to change jobs because of my tremor.	NA	N R S F A
I had to retire or take early retirement because of my tremor.		N [redacted] A
I am only working part time because of my tremor.	NA	N [redacted] A
I have had to use special aids or accommodations in order to continue my job due to my tremor.	NA	N R S F A

My tremor has led to financial problems or concerns.		N R S F A
I have lost interest in my hobbies because of my tremor.		N R S F A
I have quit some of my hobbies because of my tremor.		N [redacted] A
I have had to change or develop new hobbies because of my tremor.		N [redacted] A
My tremor interferes with my ability to write (for example, writing letters, completing forms).		N R S F A
My tremor interferes with my ability to use a typewriter or computer.	NA	N R S F A
My tremor interferes with my ability to use the telephone (for example, dialing, holding the phone).		N R S F A
My tremor interferes with my ability to fix small things around the house (for example, change light bulbs, minor plumbing, fixing household appliances, fixing broken items).		N R S F A
My tremor interferes with dressing (for example, buttoning, zipping, tying shoes).		N R S F A
My tremor interferes with brushing or flossing my teeth.		N R S F A
My tremor interferes with eating (for example, bringing food to mouth, spilling).		N R S F A
My tremor interferes with drinking liquids (for example, bringing to mouth, spilling, pouring).		N R S F A
My tremor interferes with reading or holding reading material.		N R S F A
My tremor interferes with my relationships with others (for example, my family, friends, coworkers).		N R S F A
My tremor makes me feel negative about myself.		N R S F A
I am embarrassed about my tremor.		N R S F A
I am depressed because of my tremor.		N R S F A
I feel isolated or lonely because of my tremor.		N R S F A

I worry about the future due to my tremor.	N R S F A
I am nervous or anxious.	N R S F A
I use alcohol more frequently than I would like to because of my tremor.	N R S F A
I have difficulty concentrating because of my tremor.	N R S F A

Comments:
If you have any comments, please mention them here:

Thanks for your time
